# Vaccine Hesitancy in Public Healthcare During Pandemics: An International Study to Inform Management Learning

**DOI:** 10.1002/hsr2.71190

**Published:** 2025-08-19

**Authors:** Ann Svensson, Maruška Nardoni, Anna Lydia Svalastog, Matjaž Vidmar, Helena Machado, Vanja Kopilaš, Srečko Gajović, Lucia Martinelli, Zoran Todorović

**Affiliations:** ^1^ School of Business, Economics and IT University West Sweden; ^2^ Faculty of Social Sciences University of Ljubljana Slovenia; ^3^ Department of Welfare, Management and Organisation Østfold University College Norway; ^4^ School of Engineering University of Edinburgh UK; ^5^ Interdisciplinary Centre of Social Sciences University of Minho Portugal; ^6^ Faculty of Croatian Studies University of Zagreb Croatia; ^7^ School of Medicine University of Zagreb Croatia; ^8^ Science in Society, MUSE‐Science Museum of Trento Trento Italy; ^9^ School of Medicine University of Belgrade Belgrade Serbia

**Keywords:** COVID‐19, management learning, public health, qualitative research, trust, vaccine hesitancy

## Abstract

**Background:**

This paper explores vaccine hesitancy through the lens of management learning in public healthcare during pandemics. It addresses the need for qualitative insights from active academics, focusing on their uncertainties and ambivalence regarding COVID‐19 vaccination. The study aims to deepen understanding of vaccine hesitancy during the pandemic from a management learning perspective, examining healthcare systems, governance, and community trust.

**Methods:**

Using a qualitative approach, the research draws from a multidisciplinary research network in health and digital society. A total of 27 scholars from 17 countries participated in an open‐ended questionnaire designed to elicit insights on the strategies, ethics, and public responses associated with national COVID‐19 vaccination efforts. Data collection occurred from May 2021 to July 2021, during the initial rollout of vaccines to broader populations. The analysis employed a hermeneutical framework, using thematic analysis to interpret textual data. Illustrative accounts enriched the contextual understanding.

**Results:**

The resulting themes are information and disinformation; social inclusion and exclusion; trust and distrust; individual liberties and collective constraints. The findings indicate that individual nations actions play a role in shaping public discourse, opinion, and political responses related to vaccination, despite a globalized context. The analysis reveals that historical and political factors significantly influence public health policies and perceptions of vaccine hesitancy, together with the importance of information and dialogue with various stakeholders to create trust.

**Conclusions:**

The COVID‐19 crisis, characterized by threats and uncertainties, has strained trust in public health institutions. A management learning perspective can be adopted that embraces a comprehensive understanding of the complexities surrounding COVID‐19 vaccination. By fostering collaborative learning and stakeholder engagement, public health organizations can enhance their responsiveness and build trust within communities.

## 1 Introduction

Management learning is essential for public health organizations, particularly during crises such as pandemics. It involves the continuous process of acquiring, adapting, and applying knowledge to respond to dynamic challenges effectively [1]. In times of public health emergencies, such as the COVID‐19 pandemic, healthcare organizations must engage in active learning to develop adaptive strategies that meet emerging challenges [2]. This concept of management learning underpins the ability of healthcare systems to navigate uncertainties, make informed decisions, and refine public health interventions.

One of the most pressing challenges during the COVID‐19 pandemic has been addressing vaccine hesitancy, a complex phenomenon that reflects the ambivalence and uncertainty some individuals have toward vaccination [3, 4]. The threat posed by vaccine hesitancy remains a significant public health concern [5]. As the World Health Organization identified vaccine hesitancy as one of the top ten global health threats even before the pandemic [6], the need for adaptive, learning‐oriented management strategies in public healthcare became increasingly critical. The effective management of vaccine hesitancy requires not just understanding epidemiological trends, but also comprehending the socio‐cultural, political, and economic factors that influence public attitudes toward vaccines [7, 8].

Vaccine hesitancy during the COVID‐19 pandemic illustrates how management learning in healthcare must go beyond traditional, top‐down approaches. Public health organizations need to incorporate local knowledge, continuously reassess strategies, and collaborate with various stakeholders to develop more tailored interventions [9]. Several factors significantly influence vaccination decisions, including perceived risk, government distrust, and negative healthcare experience [10, 11]. In marginalized communities, these issues are often compounded by historical injustices, necessitating the involvement of local, trusted agents to engage effectively with the population [12].

While vaccine hesitancy has been extensively examined, there is limited research on how public health systems adapt and learn to manage it during health crises. Existing studies often overlook how local trust dynamics, governance practices, and communication strategies shape vaccine uptake. This paper addresses that research gap by examining how academics interpret national responses to vaccine hesitancy, using a management learning lens to explore how uncertainty and trust‐related dynamics are perceived and navigated across different countries. This is conducted by using the situated perspectives of academics within an international research network to explore how vaccine hesitancy was interpreted across 17 different countries. This approach adds value by showing how knowledge and meaning are shaped through local experience, professional reflection, and policy environments.

This paper aims to deepen the understanding of vaccine hesitancy during the pandemic from a management learning perspective, focusing on the roles of healthcare systems, governance, and community trust. The research questions are: (1) How do academics perceive vaccine hesitancy management strategies in their countries?, (2) What factors do they identify as influencing trust/distrust in vaccination programs?, and (3) How can management learning frameworks address these challenges?

## 2 Constructivism and Actions for Management Learning

People understand the world through language, values, and social interaction. These processes are shaped by interpretation, context, and cultural meaning. From a constructivist perspective, knowledge is not fixed or objective; rather, meaning is produced and negotiated in specific social settings. Communication plays a central role in how individuals and groups navigate complexity, particularly in times of crisis, such as during a pandemic, when people are confronted with competing messages, shifting guidelines, and varying levels of institutional trust [13, 14]. In such contexts, language is not simply a medium for transmitting facts but a way of constructing shared realities and coordinating action.

As Nørreklit [15] argues, people engage in “language games” that require the integration of four interrelated dimensions: facts, possibilities, values, and communication. Facts provide the empirical basis for decisions; possibilities concern imagined futures and potential actions; values offer meaning and motivation; and communication enables legitimacy and coordination [16, 17]. If any of these dimensions are neglected, for example, when facts are presented without addressing underlying values or uncertainty, the result may be public confusion, mistrust, or resistance [18, 19]. This framework helps explain vaccine hesitancy, where rejection of official narratives often reflects not a lack of knowledge, but a misalignment between institutional messaging and people's lived experiences.

Narratives are therefore central to how communities interpret institutional actions, particularly in the realm of public health. Individuals do not absorb expert knowledge passively; they interpret and evaluate it through their own cultural backgrounds, moral frameworks, and social realities [20, 21]. For instance, vaccine communication that fails to acknowledge local concerns or community values may be dismissed, regardless of scientific merit. This highlights the need for context‐sensitive narratives that genuinely engage with the perspectives of those they aim to reach [22, 23].

The capacity to construct inclusive and meaningful narratives is closely linked to management learning, which emphasizes ongoing reflection, responsiveness, and adaptation in the face of uncertainty [1, 24]. Organizations that engage in such learning are better equipped to reassess strategies, incorporate feedback, and adapt their communication to evolving conditions [25]. In public health emergencies, these processes are essential for fostering trust and supporting informed decision‐making among diverse publics [26, 27, 28].

Collaborative storytelling supports adaptive communication, where stakeholders participate in constructing narratives that acknowledge disagreement, ambiguity, and uncertainty. This kind of meaning‐making treats trust not as a given, but as something negotiated in context [29, 30]. Narratives are not merely containers of facts; they are cultural tools that shape how collective action is imagined and enacted. Their credibility depends not only on factual accuracy but also on their alignment with community values, lived experiences, and the institutional and social environments in which they circulate [31, 32]. Vaccine hesitancy, from this perspective, emerges as a dynamic and meaning‐laden phenomenon, shaped by language, trust, and the ability of institutions to engage meaningfully with public concerns.

## 3 Research Method

### 3.1 Data Collection

The data set included 25 contributions from 27 members of the NKL (Navigating Knowledge Landscape; http://knowledge-landscapes.hiim.hr/) research network [33]. This represented convenience sampling for data collection. The data were collected from May 2021 to July 2021. At the time of the data collection period, the NKL network had about 75 members, but it is continually increasing. All members at that time were asked to be included in the study. However, those who were less engaged in the network did not show any interest in being included. The 27 members who accepted to be included contributed their viewpoints through a questionnaire aimed at mapping the rollout of the vaccination campaigns against SARS‐CoV‐2 during the early stages when vaccines were available to the general public.

All contributions are stored in a public data set, which is available with open access on Mendeley Data [33]. In accordance with the World Medical Association Declaration of Helsinki, this study adheres to the ethical principles for medical research involving human subjects. We declare that all participants engaged in this study have done so voluntarily and with informed consent (World Medical Association Declaration of Helsinki) (ethical approval: registration number SC00533).

The 27 contributors were from 17 different countries: Australia, Austria, Croatia, Czech Republic, Germany, Hungary, Italy, Norway, Portugal, Romania, Serbia, Slovenia, South Korea, Sweden, Turkey, Ukraine, and the United Kingdom (including England and Scotland). Three contributions were received from Slovenia, Sweden, and Portugal; two from Croatia and the United Kingdom; and one from each of the remaining countries. Two contributions were coauthored (from Australia and Sweden). The contributors come from different academic backgrounds, but most of them are experts in the fields of life sciences, sociology, philosophy, and medicine. The contributors were expressing their own opinions and perceptions, and their answers were not based on their fields of expertise. Their disciplinary perspectives and lived experiences inevitably informed both the shaping of the inquiry and the interpretation of the material. Rather than aiming for neutrality, the study embraces a reflexive approach, recognizing that meaning is contextually and socially constructed. The collaborative design of the study allowed for multiple perspectives to be considered during both data generation and thematic development. This plurality strengthened the interpretive process, as variations in framing and emphasis across national and disciplinary contexts helped reveal patterns, tensions, and nuances in the data.

The participants' accounts appear to be “liminal”, existing in a space between public and private discourse, as members of a research network. Within this space, ideas are continuously formed, reshaped, and redefined. What emerges is not a final or fixed answer, but rather a particular way of understanding social realities, where personal, political, cultural, and social ideas and concepts are constantly changing and contested. Hence, understandings and perceptions are not static but evolve continuously as personal reflections interact with broader public conversations. All participants were NKL researchers, mostly from Western countries, and had expertise in life sciences, sociology, philosophy, or medicine. This means the responses reflect informed, academic perspectives rather than general public opinion. That introduces a certain bias, as expert views are shaped by disciplinary knowledge and institutional contexts. While this limits how broadly the findings can be applied, it strengthens the study's ability to offer reflective and context‐sensitive insights into how vaccination policies were understood and discussed by professionals closely engaged with these issues. As members of the NKL research network and authors of this study, we acknowledge that our positions, backgrounds, and experiences influence how we interpret and analyse the data. We engaged in ongoing reflexive discussions throughout the research process to critically examine our assumptions and biases. By openly reflecting on our positionality, we aimed to enhance the transparency and trustworthiness of the study findings. The questionnaire parts were developed collaboratively within the author group through thorough discussions to ensure relevance and clarity in relation to the research aim. Although we did not conduct a formal pilot study or pre‐test, the draft questionnaire was shared and discussed extensively within the NKL network. This process allowed network members to ask questions, provide feedback, and contribute to refining the wording and structure of the questions. It is acknowledged that the absence of formal piloting and cross‐cultural validation is a limitation of the study.

The questionnaire contained three parts asking about the status and details of COVID‐19 vaccination in the respondent's country. Contributors were asked to return a short narrative text answer of 200–300 words per part. The specific questions are presented in Table 1.

**Table 1:** Questionnaire with three parts.

Part 1: The national vaccination program
Describe the COVID‐19 vaccination program in your country: what were its strengths, main weaknesses, opportunities to improve it, and threats to its success?
Part 2: Public discourse and ethics
How would you describe public responses to your country's vaccination program? What is your impression of the various collective attitudes toward the vaccination program in your country? Were there any ethical issues or concerns around the vaccine program in your country?
Part 3: Personal experience
What is your personal experience, opinion, or attitude regarding COVID‐19 vaccination?

Data saturation was not the goal of this study. The research was based on qualitative contributions from members of the NKL network, with the aim of capturing a diverse range of national perspectives on COVID‐19 vaccination. Since the focus was on gathering situated and context‐rich accounts rather than producing a fully generalizable data set, the concept of saturation is not directly applicable in this study.

The aim of this study was not to provide a systematic mapping of national vaccination programs, but rather to explore how vaccine hesitancy was experienced, interpreted, and negotiated across different country contexts. While vaccination programs undoubtedly shaped these experiences, they were neither fixed nor uniform over time. Policies evolved in response to shifting vaccine availability, changes in prioritization, political decisions, and levels of public trust or resistance. This temporal and contextual variability made it neither feasible nor analytically meaningful to treat national programs as stable units of comparison.

### 3.2 Analysis Method

To fulfill the study's aims and obtain results, thematic analysis [34] was used. This method is considered appropriate for qualitative research like our study. An inductive, or “bottom‐up”, approach of thematic analysis was used, not driven by theoretical interest. The themes were found without trying to fit into a pre‐existing coding frame. Thus, the thematic analysis has been data‐driven, and the themes identified were strongly linked to the collected data [34]. However, the coding process could not avoid a pre‐existing coding frame, and our analytic preconceptions, as the authors cannot free themselves of their theoretical and epistemological commitments. The thematic analysis was primarily conducted by the first three authors, who worked closely together to ensure consistency and depth in interpretation.

For the purposes of thematic analysis, we emphasized variations in vaccine hesitancy, as well as politics and safety trust and distrust in the different countries. The thematic analysis included a number of steps and levels, which were performed iteratively and in parallel [34]. In phase one, the entire data set was read through several times before the coding started, to become familiar with all aspects of the data. Then, in the second phase, initial codes were generated, together with an initial list of ideas about what was identified in the data, in relation to the aim of this paper. Specific accounts from the participants' descriptions were also identified. In the third phase the analysis focused on a holistic perspective in relation to the whole data set, and potential themes were suggested. In phase four, the themes were determined and reviewed to include coherent and meaningful data and to include clear and identifiable distinctions between the identified themes. The fifth phase included the refinement and the final naming of the themes. Furthermore, the data within the themes were analysed in this phase to identify the essence of each theme. The sixth and last phase involved the final analysis and writing of the findings. The last phase also included reviewing the data again using the themes as a tool to ensure that all aspects were considered as a way to verify the findings. As such, the process continued moving between the different phases in searching and reviewing of themes, until the final analysis [34]. The themes capture important content in relation to the research question and represent the meaning within the data set.

The analysis was shared with all co‐authors, who reviewed and provided feedback on the findings and thematic structure. This collaborative review process functioned as a form of internal triangulation across disciplinary and national perspectives. While we did not apply formal peer debriefing or member checking, the iterative exchange within the research team contributed to the credibility of the findings. Thus, coding and theme development were conducted collaboratively to minimize individual bias and to ensure that multiple perspectives were considered. The collective and iterative nature of the discussions helped surface divergent perspectives and challenge assumptions within the group, strengthening the interpretive validity of the findings. The authors were not external observers but part of the same community as participants in the study. All authors engaged in ongoing reflexive discussions throughout the analysis process, critically examining assumptions and interpretations. Moreover, all data are available in a public repository [33], which supports transparency and allows for external scrutiny of the material and interpretations. To enhance the trustworthiness of this study, the analytical process was documented thoroughly, and the authors were engaged in reflexive discussions to address potential biases. These steps helped ensure the credibility and rigor of the findings. The findings from the analysis were discussed from a constructivist and management learning perspective to inform theory.

In the findings section, we provide the analysis with the following four themes: (1) information and disinformation; (2) social inclusion and exclusion; (3) trust and distrust; (4) individual liberties and collective constraints. The findings include rich descriptions, with illuminative accounts from the entire data set. The accounts, from the 27 participants, were chosen as to be representative and typical in relation to our purpose and refer to country‐specific observations that illustrate the key themes identified in the data. These accounts are not intended to be representative in a statistical sense, nor are they chosen as extreme or outlier cases. The selection aimed to avoid bias by including experiences, both positive, negative, and ambivalent, related to vaccination, communication, and trust. The goal was not to generalize, but to give empirical grounding to the thematic analysis.

### 3.3 Findings

Participants' accounts revealed an assemblage of perspectives that reveal the complexity of social, cultural, political, and emotional elements related to perceptions of risks and benefits of COVID‐19 vaccines and vaccination programs.

A prominent aspect was the elaboration of fluid dichotomic categories, namely between those who accept vaccination and those who refuse or hesitate, despite the fact that most participants recognize the difficulty in making “categorical” decisions (e.g., acceptance vs. hesitancy or refusal) in a context marked by uncertainties in the present and for the future. In the testimonies collected in the qualitative study, the main dichotomous categories that emerged were the following: (1) information/disinformation, (2) social inclusion and exclusion, (3) trust/distrust, and (4) individual liberties and collective constraints. However, these broad categories encompass many different questions (of an ethical, pragmatic, and political nature) that apply both across the categories but also within these categories.

### 3.4 Information and Disinformation

In general terms, poor information or poor communication is identified as one of the main factors for hesitation or refusal of vaccination. However, this view is not unanimously shared by the participants in the study, as some of them report that, on the contrary, vaccine hesitation occurs among social groups that are more actively seeking information about vaccines. The perspective of the need to better inform populations to prevent attitudes of refusal or hesitation to vaccinate, manifested by the participants, is dual: some of them express a position that emphasizes the need to “educate the public”, while others prefer listening to populations and actively promoting public engagement.

The following accounts exemplify well the idea that the public should be informed and “educated” about the benefits of vaccination, and governments, together with the media, have sometimes failed in their roles of providing clear and timely information:

Weaknesses [of vaccination programs] have been and still are mostly connected with insufficient information provided by the government and media to the public. (…) Since that time situation improved in distribution of correct information and better explanation of scientific background of vaccine development. Many experts explained in the media everything correctly to the public. It seems that now more people believe them.

(L.L., Czech Republic)

Overall, the greater the perception of having good information on vaccines, the greater the willingness to vaccinate. (…). The level of education, in Italy, seems to be the variable that has the greatest influence on the intention to want to be vaccinated: the lower the level of education, the greater the willingness not to be vaccinated.

(L.M., Italy)

The percentage of vaccinated health workers and cohorts that have been less represented so far should be increased, which can be achieved through better education and information.

(Z.T., Serbia)

The most common reasons to not be vaccinated is that people are afraid of side‐efffects, they do not believe that a vaccine is safe enough, and that they do not believe that COVID19 is dangerous for one's health.

(A.S., Sweden)

Other participants emphasized the need to engage the public in decision‐making and promoting empathy with local communities. For example, one participant from Norway highlighted the benefits of engagement of all relevant stakeholders before implementation of vaccination programs. In her view, the deputy director of health's previous military experience was particularly beneficial for crisis management, as it made him aware of the importance of collaborative, engaging, and shared decision‐making processes:

[ensuring] that as many voices as possible are included in the planning, to secure a joint understanding of the situation and key goals, and then follow up with renewed strategies and goals as the process unfolds. This secured a broad discussion of strategies in advance of decision making, public communication, and implementation (…). In addition, the public and dynamic debates between national government and local governing bodies that has shown how local voices matters.

(A.L.S., Norway)

In addition to challenges of adequate provision of information, two categories emerged in the arena of accounts on vaccine hesitancy we could find in the qualitative study: the “disinformation” promoted by social media and the anti‐vaxxers, and the “cultivated public” who declines vaccination due to access to scientific knowledge. The following accounts highlight experiences with anti‐vaccination campaigns gaining expression on the Internet and social media.

Intense anti‐vaccination campaigns, anti‐mask campaigns in social media and on the internet.

(I.C., Romania)

There are groups of people who debate on social media about the dangers of vaccinations, and especially the vaccines which are developed fast. Conspiracy theories about vaccines as a means to control the world have been expressed.

(S.L. and M.T., Sweden)

More negative discourse does arise, in particular on social media. Unlike countries such as the USA pro and anti‐vaccination discussion is not split along political lines. Instead, anti‐vaccination discourse centers on (mis)information about the current licensed vaccines (for instance, stating they have not been tested fully and are unproven) or vaccinations in general (that they seek to control populations, are designed to profit big pharma etc.).

(H.W., UK)

There is a small minority of individuals who still believe that this pandemic is a hoax, and that the vaccination is a great plan of Bill Gates to control and track us all.

(V.K., Croatia)

### 3.5 Social Inclusion and Exclusion

In several accounts, the participants framed hesitancy as an epiphenomenon of “distance to science”, namely among disadvantaged and minority groups. Several participants highlighted the necessity of governments being sensitive, in their communication roles, to concrete concerns of an extensive diversity of socioeconomic groups with different needs and backgrounds, and the need for addressing both the benefits of vaccination and the reasons for doubts.

Slovenian political institutions, which are responsible for implementation of public policies did not succeed to find adequate communication strategies with public. They did not succeed successfully promote the importance of vaccination for all aspects of human life. For example, to be successful by promotion of vaccination of various strata of population, I imagine that the state's institutions would need to be sensible for different populations' specific concerns, for possible previous experiences concerning some other type of vaccination, for the fact that there exist various religious, political and socioeconomic affiliations of various strata of public. The worst strategy is to communicate with the public in an underestimated way.

(F.M., Slovenia)

(An) improvement of the vaccination process would be a simple and consistent public campaign promoting positive effects of vaccination while addressing the reasons for hesitancy and scepsis towards vaccines.

(M.N., Slovenia)

Certain religions or ethnically distinct groups who are already excluded from access to the health system were described as the most prone to experience vaccination hesitancy. The following accounts manifest the need to elaborate communication campaigns targeting ethnic or religious minorities.

More broadly, uptake of the vaccination amongst some ethnic minorities has been slower than the general population; perhaps due to the historic mistreatment of these groups by medical systems and state institutions in general, members of these demographics are less likely to feel they can trust the vaccination program. To counter this, the NHS has produced public information campaigns featuring celebrities from Black British and Asian backgrounds to encourage members of these groups to take up the opportunity to be vaccinated to protect themselves and others in their communities.”

(H.W., UK)

Vaccination continued to be hampered by poor communication to people from different cultural groups. Such failures in communication contributed not only to the spread of the disease but a continuing fear of being vaccinated.

(R.C. and K.S., Australia)

Other participants pointed out the influence of religion as a key factor for refusal and/or hesitancy among some communities.

The weakest success of vaccination was (and still is) in the Sandzak region, where most of the Muslim Bosniak population lives. By all accounts, there is a fear of vaccine safety and a belief that COVID will overcome on its own.

(Z.T., Serbia)

Interestingly, some vaccination opponents linked their fears to Islamic codes of conduct. For example, during the month of Ramadan, fears were raised that vaccination during the fasting period was inadmissible. The Presidence of Religious Affairs, Presidency of the Republic of Turkey (DITIP) recently issued a Fatwa on the subject, stating that, The issue of whether vaccines and injections will invalidate the fast is not included in the scriptures, and (…) a direct provision regarding the effect of vaccination on fasting is not found in our classical sources.” It was concluded that there was no concern about this with regard to vaccination.

(M.Ş., Turkey)

### 3.6 Trust and Distrust

In our qualitative study, we could find diverse references to trust and distrust that amplify and provide a more detailed understanding of these topics. Fears about side‐effects and concerns about lack of effectiveness were often reported by the participants, although the overall tendency, in balancing risks and benefits, was vaccine acceptance. Some of the participants expressed general concerns about vaccine safety or serious side effects, while others manifested concerns over particular brands of vaccines.

The potential ineffectiveness of the Chinese vaccine has caused serious social tensions.

(N.B., Hungary)

The difficulties in securing a vaccine were exacerbated by reports of the risks involved with the AstraZeneca Vaccine and a population that was increasingly hesitant to get vaccinated (…) fears about the long‐term side effects, and changing DNA.

(R.C. and K.S., Australia)

My direct family members experienced no complications from vaccine intake. However, some friends and acquaintances did. In some cases, the consequences were quite drastic to the point they opted for not taking a second dose after medical advice.

(C.F., Portugal)

Reports about rare serious side effects of vaccine have caused great worry among some persons, leading to refusal to take a certain brand of vaccine and verbal abuse of the health care staff in some instances.

(S.L. and M.T., Sweden)

But the main reasons lie in concern about possible side effects, in the fact of not belonging to high‐risk categories, and lack of confidence in the validity of the process that led to approval.

(L.M., Italy)

A minority of the participants point to the fact that COVID‐19 vaccines represent certain technological advances that have not yet been tested, which makes them fearful about their risks. One of the participants expresses her fears in this regard as follows:

The mankind is indebted to scientists, governments, and private sector for the provision of vaccines against a new virus in such a short period. However, the facts that a new drug has never before emerged in the market so quickly and these are the world's first gene‐engineered vaccines might be disquieting. Also, the current vaccination process worldwide can be treated as an ongoing clinical trial that predictably will bring new knowledge, resulting in the change of currently established vaccination schemes and recommendations. At the same time, we do not have other choice as to get vaccinated and to accept that the benefits of vaccination significantly overweigh its risks.

(C.N., Ukraine)

One aspect that stood out in relation to the themes of trust or lack of trust was the relationship between national governments and their citizens. From the perspective of some participants, one of the main reasons for vaccine hesitation is the historical, social, and cultural context of their country, which leads people to fear that their governments do not always act with the best of intentions.

One of the participants articulated the low confidence in the effectiveness and safety of the vaccines against COVID‐19 in his country, with a general, historically prevailing, tendency of lack of public trust in so‐called rational institutions (and procedures). In his words:

The pandemic has triggered widespread disinformation that has undermined both understanding and acceptance of science and public policy. Historical “relics”—if we use the concepts of Polish sociologist Pijoter Sztompka—are still playing an important role in such transitional countries as it is Slovenia. There is lack of historically based mechanisms which help to construct trust in various social subsystems (including health subsystems). This means that there is still a lack of culture of trust based on factors such as prevalence of rational discourse over irrational discourse, transparency with regard to the openness of communication between laymen and experts, etc.

(F.M., Slovenia)

Other participants considered that the lack of trust in the government and national vaccination programs was due to a complex interrelation between several elements: from shortage of vaccine supplies in the early stages of vaccination rollout to a generalized sense of lack of trust in the government's actions. The latter were based on a spectrum of reasons, spanning from suspicions of corruption to perceived limitations of human rights and freedoms (lockdowns, social distancing, facemasks, and other restrictive measures for longer periods than advised by scientists and health experts). The following accounts evidence the diverse perspectives on the lack of trust in political actors to manage the pandemic in ways that are transparent and accountable:

The public response to the vaccination program in Germany was and still is very mixed and characterised by a lot of scepticism, mainly because of the slow distribution of the vaccines and sometimes just horrible communication from the government regarding vaccines, lockdown, and other restrictions. Most people in Germany doubt that the government will be able to follow through on its vaccine campaign goals. Restrictions, vaccination prioritisation, and the ways to get an appointment for a Covid vaccination currently vary a lot across Germany's 16 states and sometimes even from city to city. This creates an array of rules that can make it difficult for people to know what is currently allowed in their regions or who is eligible for and how to get an appointment for the COVID‐19 vaccination.

(A.P., Germany)

A major discussion at the outset concerned the fact that Turkey was purchasing Sinovac vaccines from China only—and also too late. At the same time, it was being reported in the media that vaccination tourism was already taking place and that citizens were being vaccinated with Biontech vaccines, paying privately. This gave rise to speculations that the country was not doing enough for all its citizens. In the meantime, however, Turkey was negotiating with Biontech, whose key players were of Turkish origin. This sparked discussions that Turkey had enormous problems conducting science and technology at a high level and that there was a brain drain.

(M.Ş., Turkey)

Slovenia faced a lack of vaccine shots in its early phases of national vaccination programme, that have may deepened the negative stance towards official authorities, while the AstraZeneca scandal (e.g., the company failing to deliver vaccine shots by the due date) achieved a similar effect. Correspondingly, this had crucially aggravated trust in available vaccines at the time. Another thing worth mentioning regarding the erosion of trust is the prior discontent with the governmental restrictive measures and its law‐making uptake, which proved to be too long‐lasting and was often in dissonance with the recommendations of epidemiologists and other health experts.

(M.N., Slovenia)

Positive accounts of government actions and their relationship to the roll‐out of the COVID‐19 vaccines were also reported by some of the participants in the survey, while some of them expressed more mixed feelings:

My impression on the various collective attitudes towards the vaccination: high levels of public trust and confidence (science, health care system, task force), that make people feel safe and protected, devaluing the risks and preventive measures.

(S.S., Portugal)

By the beginning of Summer 2021, nearly half of the Scottish (and UK) population is fully (double) vaccinated, and there has been little resistance to mass vaccination in public discourse. Anti‐vaxxer movements are sparse and are not gaining much attention—across all age groups, people are keen to get vaccinated and share images of appointment invitations and visits to the mass vaccination centres on social media, etc. (Blue envelopes in which invitations are sent through the post are becoming a cultural reference point.) Most of the concern expressed publicly was directed at the speed of the clinical trials and approval process, which was refuted under the narrative of extensive (public) investment and almost exclusive R&D focus of large and resourceful pharmaceutical companies (unlike in “ordinary” vaccine development).

(M.V., Scotland)

Here, people often make positive comments about how quickly the rollout has been achieved and how reassured they feel to know that so many people in the population have been immunised. The vaccination clinics are generally very full, showing that people are taking up the opportunity to receive their vaccinations, and the walk‐in clinics have been very busy. On some occasions, anti‐vaccination protestors have arrived at the centre and attempted to persuade people there not to get vaccinated, but their efforts have been largely unsuccessful.

(H.W., UK)

Finally, vaccine hesitation or refusal, or even anti‐vaccine movements, were seen as political actions in which citizens expressed their negative assessment of the enthusiastic political rhetoric about vaccination.

The persuasive political and medical discourses are accompanied by the enthusiastic argumentations in favour of science against conspiracy theories and vaccination scepticism in social communities. Yet it seems that the criticism towards the governing political right has influenced the citizens will to get vaccinated. There is a lot of hesitation, especially in young people. The special promotional measures, for example, that one could choose the vaccine brand and the option to get vaccinated in the public areas, have not shaken the prevailing restraint yet.

(R.Š., Slovenia)

Public responses to the vaccine in Austria are politically charged and risk to (re)produce societal fault lines. First, vaccine hesitancy is widespread across different parts of the population in Austria, and has increased over the last years, also in regard to standard vaccines such as measles (…). Common explanations for vaccine hesitancy in Austria are a strong emphasis on “the natural” (…), and a legitimacy crisis of political elites. In public discourse, attitudes towards vaccines became the symbol for (dis)agreement with governmental measures to contain the virus. Being an “anti‐vaxxer” marked a red line in criticism about pandemic politics.

(W.S., Austria)

Interestingly, many participants also expressed the feeling that perhaps the willingness to be vaccinated was more related to the generalized desire of coming back to normal life (before the pandemic) and re‐gaining individual freedom overcoming the need for collective constraints, than mere confidence in the COVID‐19 vaccine safety and effectiveness. This balance between the individual and the collective leads to complex ethical questions that lack broader debate, as is analysed in the next section.

### 3.7 Individual Liberties and Collective Constraints

The large majority of the participants reported their feelings that the population in their respective countries were willing to get vaccinated to gain individual liberty to travel or not to be subjected to restrictive measures applied to unvaccinated people. The following participant refers to more liberty for vaccinated people, and to financial compensation to those willing to be vaccinated:

South Korea bans private gatherings of more than four people, and families can only gather up to eight people. However, from June 1, those who have been vaccinated can gather up to eight or more family members. Starting in July, vaccinators will not be obliged to wear masks outdoors. Major public facilities also offer vaccinators discounts or exemptions from admission or usage fees. So many are actively participating in vaccinations.

(J.S., South Korea)

Many of the accounts referenced the influence of “wanting to get back to normality” in attitudes towards COVID‐19 vaccines:

Norwegians fly more than most countries. Statistics from EU show that from second quarter of 2020, all countries but they had a 95% or more drop in number of passengers, Norway had the least with—89.7%. Thus, travelling and the need for a corona passport should also be included when discussing vaccination.

(A.L.S., Norway)

Most people are very anxious, they want to be vaccinated as soon as possible, as they believe it's a “miracle” that avoids infection and opens the opportunity to have a “normal” life.

(S.S., Portugal)

Willingness to get vaccinated as soon as possible is typical for young and socially active people, eager to return to “normal” but can be treated also as a manifestation of their high level of education and personal culture.

(C.N., Ukraine)

There has been a lot of positive discourse around the vaccination programme in the UK. The speed with which the rollout has unfolded has been reported positively in the media and connections made between getting a vaccination and gaining more ‘freedoms’ through the end of lockdowns and the ability to travel overseas.

(H.W., UK)

The reliefs it has for social life (access to services such as, for example, going to the hairdresser, restaurants, events, etc., for tested, vaccinated, and recovered people).

(W.S., Austria)

In the opinion of some of the participants, the optimistic stance over COVID‐19 vaccines as a means to get back to normality is one of the reasons why ethical aspects of vaccination rollouts are not frequently debated. While in the first stage of vaccination programs, there was some public debate about the criteria for priority, the definition of risk categories of people, and unequal access and differences between regions or countries, as time went by and the population's vaccination coverage increased, the ethical debate faded or lost its intensity in the public space. As expressed by one of the participants:

The ethical problems associated with COVID‐19 vaccination are: potential discrimination against those who refuse to be vaccinated; changing priority criteria without public consensus and in the absence of accountability and transparency; messages from the Government that lead to the misconception that this campaign will allow economic and social normality to resume in September (…) there is an urgent need to clarify publicly that vaccination is just one measure—certainly important—in the management of the pandemic. But even more important is to ensure that the entire population has access to health care and to require governments to develop preventive measures related to pandemics, and to ensure that access to health is effectively guaranteed to all who need it.

(H.M., Portugal)

In the following words of one of the participants, the prominence of individualism is preventing a broader, open, and transparent ethical discussion on the implications of COVID‐19 vaccines:

There is a strong emphasis on the individual perspective; lack of concern about inequities and disparities (local, national, and global); too much hope on the short‐term, with no discussion about the future; lack of criticism about the social responsibility of pharmaceutical industries. COVID‐19 vaccination is helpful, but it is not the “miracle” or the “magic bullet” that a lot of people are expecting. Expectations and benefits should never mask limitations and risks—public awareness on these topics is essential.

(S.S., Portugal)

Other participants point out that while ethical aspects of vaccination for other diseases are discussed, apparently the debate is scarce when it comes to COVID‐19 vaccines: limited, for example, to criteria for priority, people providing false information to get quicker access, or inequality between countries in accessing vaccination stocks:

Vaccinations in general often create a discussion about voluntariness versus obligation, about justice, solidarity, and participation. It is also about what trust people have for healthcare, medical science, and public health authorities, and for information and media. However, when it comes to vaccination against covid‐19, these more ethical issues do not seem to be so much discussed. However, discussions have arisen regarding the fact that some people try to get ahead of the order of priority, when they claim that they are in a risk group without being.

(A.S., Sweden)

There were two instances when the ethical issues concerning vaccination were at the fore of public discourse – at the time when one vaccine was linked to rare, but serious side‐effects, and as part of a dispute with the European Union concerning the supply of vaccines and their ingredients. In fact, this is in stark comparison to less physically invasive but more socially disruptive public health measures, such as mask‐wearing and social isolation. There the debate has been intensifying as time went on noting the increasing impatience and resentment of the restrictions imposed.

(MV, Scotland)

## 4 Discussion

Vaccine hesitancy poses a significant challenge to public health, particularly during crises like the COVID‐19 pandemic. The World Health Organization (WHO) identified it as a key threat to global health, emphasizing that addressing this issue requires an in‐depth understanding of the social, psychological, and cultural factors that shape public attitudes [6]. Recent studies have shown that public trust in institutions, the role of misinformation, and perceived risks of vaccination are crucial determinants of vaccine acceptance [35]. This study revealed the limitations of top‐down public health strategies and emphasized the need for adaptive management‐learning approaches.

Vaccine hesitancy during the COVID‐19 pandemic presented significant challenges for public health institutions, particularly regarding how to communicate risk and build trust. The study identified poor information or communication as a factor contributing to vaccine refusal. It pointed to early gaps in public communication, where governments and media failed to provide clear, timely, and coherent messaging. From a management learning perspective, this reflects a lack of anticipatory adaptation, where institutions did not yet have processes in place to learn from public feedback, adjust messaging strategies, or respond to evolving concerns in real time [1, 25]. However, the notion that low vaccine uptake was only a result of insufficient information was also challenged. Vaccine hesitancy also emerged among highly informed individuals, those who actively sought out information, scrutinized scientific claims, and made deliberate decisions based on personal assessments of risk. This highlights a critical insight from constructivist and management learning theory, as knowledge is not simply transmitted from experts to the public, but interpreted through existing belief systems, lived experiences, and social values [13, 14].

The tension between individual freedom and collective responsibility found in the study points to an ethical dimension often overlooked in traditional management models. Effective management learning requires incorporating ethical reflection alongside empirical data to better understand motivations behind behaviours like vaccine refusal. This broadens the capacity of management to develop communication strategies that resonate not only cognitively but also morally with diverse populations. The critique of inadequate and insensitive communication points to limited organizational management's reflection and adaptation. Management that fails to listen and incorporate feedback from diverse publics does not engage in the iterative learning necessary for effective crisis management [1, 24, 36]. This is compounded by dominant narratives framing vaccination as a personal freedom issue, marginalizing questions of social justice, equity, and corporate responsibility, central to ethical management learning [31]. The lack of open, transparent ethical debates curtails opportunities for collective reflection on the societal impact of vaccination policies and constrains the development of trust as a negotiated, context‐dependent phenomenon. Research suggests that balancing these ethical concerns requires sensitive, culturally informed strategies that respect individuals' rights while promoting the public health benefits of vaccination [37]. The episodic nature of public ethical discussions, peaking during vaccine side effect controversies or supply disputes, illustrates reactive rather than proactive learning processes. This pattern reflects challenges in embedding continuous and anticipatory learning mechanisms. Without sustained attention to ethical complexities and stakeholder perspectives, organizations risk repeating mistakes and losing crisis resilience [2, 38].

Vaccine hesitancy among disadvantaged and minority groups was shown to be related to a “distance to science”, revealing how trust and distrust around COVID‐19 vaccines arise from complex socio‐political and cultural factors. This highlights the importance of communication that responds to diverse socioeconomic, religious, and cultural contexts [39]. This illustrates the constructivist idea that meaning is negotiated in social settings and that communication is not just the transmission of facts but the construction of shared realities [13, 14]. The described information campaigns involving minority representatives illustrate collaborative storytelling, where narratives are co‐constructed to reflect community values and lived experiences [29, 30]. This approach treats trust as negotiated, not given, requiring communication to be credible both factually and culturally [32]. In crisis management, the ability of management to recognize and adapt to these varied knowledge practices is central to learning and strategic responsiveness.

Two distinct orientations toward public communication were evident in the study. One emphasized the need to “educate the public” by improving scientific literacy and promoting trust in expert authority. This reflects a more traditional, top‐down model of health communication. Others called for a more dialogical approach, where the public is seen not as passive recipients of information, but as active participants in the process of meaning‐making. This latter view is closely aligned with management learning, which stresses the importance of listening to multiple voices, integrating feedback, and adjusting institutional practices based on interaction and reflection [24]. Concerns about vaccine side effects and brand‐specific scepticism show that factual information alone is insufficient to ensure vaccine uptake. This aligns with constructivist insights that facts must be integrated with values, communication, and possibility to create meaning [15]. Management learning demands recognition of these multiple dimensions when designing communication strategies, emphasizing adaptation to varied public concerns.

The study suggests including diverse stakeholders, such as local health professionals, community leaders, and municipal authorities, to better ensure broader engagement and responsiveness in vaccination strategies. This kind of collaborative planning aligns with Weick's [16] view that sensemaking in organizations depends on inclusive processes that allow for ambiguity, revision, and continuous negotiation of meaning. This includes the ability to involve multiple actors in dialogues, co‐decision‐making, and ensure coordination across levels of governance [40], illustrating how learning‐oriented leadership can enhance institutional legitimacy and effectiveness under pressure. Management learning thus involves creating spaces for dialogue where disagreements and uncertainty are openly addressed [29]. This collaborative storytelling enables a dynamic sensemaking process crucial for coordination in crises. Failure to engage with diverse public voices risks perpetuating resistance and misinformation, which the study exemplifies through reports of distrust rooted in historical injustices or government opacity.

The study reveals how alternative narratives, circulating mainly via social media, interacted with institutional communication. It was observed that misinformation, conspiracy theories, and generalized scepticism about vaccines gained traction in part because institutional responses were perceived as inconsistent, overly directive, or detached from public concerns. From a management learning standpoint, these failures emphasize the need for reflexivity in communication, where institutions not only disseminate messages, but also reflect on how those messages are received, what assumptions they carry, and how they align with or contradict public values [15, 19]. Another notable aspect concerned a “cultivated public” who refused vaccination not due to misinformation but based on critical engagement with scientific data and political narratives. Their hesitancy was rationalized through personal ethical positions, previous negative experiences with healthcare, or distrust in state motives. These responses challenge the assumption that correcting knowledge deficits alone can change behaviour and point instead to the importance of narrative legitimacy, namely, whether public health narratives are seen as credible, inclusive, and responsive [30]. The study shows that institutional success in navigating vaccine hesitancy depends not just on expertise or authority, but on management's capacity to learn in action, to reassess strategies, understand social dynamics, and incorporate local knowledge and dissent [41]. Communicative adaptability, stakeholder engagement, and iterative learning are essential practices for building durable public trust in times of crisis [42].

The diversity of public reactions across countries, influenced by past experiences, political climates, and cultural values, reinforces that learning in management is not a one‐size‐fits‐all approach. Rather, it involves continuous adaptation to emergent realities and stakeholder input. For instance, recognizing that vaccine hesitancy may be linked to political opposition or collective ethical dilemmas suggests that communication cannot only target scientific facts but must also engage narratives that acknowledge social identities and tensions [22]. The study highlights individual freedoms like travel and service access, while largely overlooking collective and ethical aspects of vaccination. This imbalance weakens trust by disconnecting messaging from community values and lived experience. Incentive‐based approaches, like offering vaccinated individuals greater liberties or financial compensations (as described by the South Korean participant), reflect a transactional communication rather than collaborative storytelling processes [29]. The study also points to a fragmented landscape of communication and policy application across countries and even within nations, with inconsistencies in lockdown measures, vaccine prioritization, and access rules. This fragmentation complicates the social construction of shared realities and undermines collective action [25]. From a management learning standpoint, such incoherence reflects insufficient coordination and integration of multiple stakeholder perspectives, impeding the development of cohesive strategies capable of dealing with complexity in crisis contexts.

## 5 Limitations

The study has several limitations. All participants were recruited from the Navigating Knowledge Landscapes (NKL) network. Although they represent a variety of disciplines, mainly in medicine, sociology, philosophy, and the life sciences, and come from multiple countries, they do not reflect the general population. Their professional roles and academic backgrounds may have influenced both their interpretations and how they chose to present them. Moreover, the findings are based on self‐reported narratives, which may be shaped by selective memory or social desirability.

The data were collected between May 2021 and July 2021, during the early phase of COVID‐19 vaccine rollout. At the time, vaccine policies, availability, and public attitudes were changing rapidly. These conditions limit the relevance of the findings to later phases of the pandemic or to different socio‐political environments.

The thematic analysis was mainly conducted by three of the authors. While the full author group was engaged in reviewing the results and shaping the final interpretation, no formal procedures such as member checking or external validation were used. Relying solely on internal consensus may reduce the study's qualitative rigor.

The aim of the study was not to describe national vaccination programs, but rather to explore how academic participants interpreted vaccine‐related debates within their own contexts. This focus provides analytical insight but excludes the perspectives of the general public and healthcare workers. Consequently, the findings are more interpretative than experiential.

Finally, the NKL network is predominantly European, which may limit the diversity of perspectives and reduce the transferability of the results to non‐European settings with different cultural and healthcare systems.

## 6 Conclusions

This paper aimed to deepen the understanding of vaccine hesitancy during the pandemic from a management learning perspective, focusing on the roles of healthcare systems, governance, and community trust.

This study demonstrates that vaccine hesitancy cannot be resolved through facts alone. Public trust depends on how language, values, and actions align with lived experiences and institutional credibility. Messaging focused narrowly on individual freedom, such as travel or access incentives, undermines trust when communities perceive neglect of collective ethics, fairness, and transparency. Mistrust in authorities emerged as a critical barrier. Historical scepticism toward government intent and inconsistent communication, especially during supply shortages or shifting eligibility criteria, eroded legitimacy. Failures to engage proactively with public concerns, coupled with reactive responses only during controversies, reveal weak organizational learning systems. Management lacked feedback mechanisms to anticipate evolving doubts and adjust dialogue before trust declined. The distributed role of local actors surfaced as a key insight. Where health professionals, community leaders, or municipal authorities were involved in co‐designing messaging and planning delivery, trust improved. This emphasizes that effective vaccine communication relies on decentralized, culturally sensible actor networks rather than top‐down campaigns alone.

The study emphasizes the need for anticipatory, iterative, and context‐sensitive learning. Organizations must integrate empirical facts with values, opportunities, and culturally resonant communication. Success depends on adaptive leadership capable of listening to diverse voices, coordinating across governance levels, and responding in real time. This study advances management learning theory by showing how narratives and meaning‐making processes shape vaccine hesitancy. It reveals that trust‐building during crises requires more than technical expertise; it demands ethical responsiveness, communicative reflexivity, and stakeholder engagement. Management teams that navigate uncertainty effectively are those that learn in action, engaging communities continuously, responding to cultural and historical contexts, and proactively rebuilding legitimacy for shared public health goals.

## 7 Practical Implications

The findings from this study indicate that effective public health communication during pandemics requires more than campaigns designed to persuade people to get vaccinated. Instead, there is a need for ongoing, structured opportunities where people can openly express concerns, questions, and even opposition without being dismissed or stigmatized. Vaccine hesitancy should not be treated solely as misinformation or irrational behaviour, but understood as a response influenced by past experiences, levels of institutional trust, and social and cultural contexts.

In practical terms, this means inviting a broader range of voices—especially those who are sceptical or hesitant—into the development of communication strategies. Public health agencies should collaborate with community representatives, educators, and civil society actors to design messaging that reflects local concerns. Health professionals should also receive training that helps them handle disagreement respectfully and engage in dialogue without judgment. Additionally, communication should acknowledge uncertainty when it exists, rather than presenting overly confident or simplified messages that risk eroding trust.

These practices may not produce immediate increases in vaccine uptake, but they can build longer‐term confidence in public health systems and foster a sense of shared responsibility. By treating communication as a two‐way process rather than a one‐sided campaign, public institutions can become more responsive, transparent, and resilient in the face of future health crises.

## Author Contributions

**Ann Svensson:** conceptualization, investigation, writing – original draft, methodology, validation, visualization, writing – review and editing, formal analysis, data curation. **Maruška Nardoni:** conceptualization, investigation, writing – original draft, methodology, validation, visualization, writing – review and editing, formal analysis, data curation. **Anna Lydia Svalastog:** conceptualization, investigation, writing – original draft, methodology, validation, visualization, writing – review and editing, formal analysis, project administration, data curation. **Matjaž Vidmar:** conceptualization, investigation, writing – original draft, methodology, validation, visualization, writing – review and editing, formal analysis, data curation. **Helena Machado:** conceptualization, investigation, writing – original draft, methodology, validation, visualization, writing – review and editing, formal analysis, data curation. **Vanja Kopilaš:** conceptualization, investigation, writing – original draft, methodology, validation, visualization, writing – review and editing, formal analysis, data curation. **Srečko Gajović:** conceptualization, investigation, writing – original draft, methodology, validation, visualization, writing – review and editing, formal analysis, data curation. **Lucia Martinelli:** conceptualization, investigation, writing – original draft, methodology, validation, visualization, writing – review and editing, formal analysis, data curation. **Zoran Todorović:** conceptualization, investigation, writing – original draft, methodology, validation, visualization, writing – review and editing, formal analysis, data curation.

## Ethics Statement

This study has been conducted according to the principles expressed in the Declaration of Helsinki, referring to the ethical principles for medical research involving human subjects [43]. According to the study protocol, it did not collect any sensitive information, according to the Declaration of Helsinki.

## Consent

All participating human subjects provided their written consent prior to study participation. The ethical approval registration number is SC00533, University of Edinburgh, Scotland. Consent for publication has been obtained from the individual persons.

## Conflicts of Interest

The authors declare no conflicts of interest.

## Transparency Statement

The corresponding author, Ann Svensson, affirms that this manuscript is an honest, accurate, and transparent account of the study being reported; that no important aspects of the study have been omitted; and that any discrepancies from the study as planned (and, if relevant, registered) have been explained.

## Data Availability

The data set generated and analysed during the current study are available in the Mendeley Data repository, https://data.mendeley.com/datasets/bwdf6v5wgr/1.
